# It tastes sweeter when melted: Exploring the impact of food temperature on tongue temperature and perceived sweetness/vanilla

**DOI:** 10.1016/j.sctalk.2025.100424

**Published:** 2025-03

**Authors:** Hannah McNeill, Rebecca Ford, Ian Fisk, Margaret Thibodeau, Gloria Liu, Marion Doyennette, Qian Yang

**Affiliations:** a School of Biosciences, University of Nottingham, Nottingham LE12 5RD, United Kingdom; b Unilever R&D Colworth Science Park Sharnbrook, MK44 1LQ, United Kingdom; c Unilever Foods Innovation Centre Wageningen, Plantage 14, Wageningen, 6708, WJ, the Netherlands

**Keywords:** Thermal imaging, Sweetness perception, Tongue surface temperature

## Abstract

The relationship between perceived sweetness intensity and temperature of food is complex. Previous research on the effect of temperature on sweetness perception primarily focused on single solutions. This study aimed to address the gap by using an infrared camera to measure tongue surface temperature, explore tongue temperature ranges, the relationship between sweet/flavour and tongue temperature at different serving temperatures during real food consumption. Participants (*n* = 22) consumed custard served at warm (59.1 ± 0.8 °C), ambient (24 ± 0.6 °C), chilled (4.6 ± 0.5 °C), and frozen (−2.7 ± 0.3) temperatures. An infrared camera was used to capture participant tongue surface temperature. Sweetness and vanilla intensity were recorded using a modified General Labelled Magnitude Scale. This study demonstrated that infrared imaging could effectively capture tongue surface temperature. Results revealed tongue surface temperature recovered to baseline more efficiently after cooling than warming. A weak positive correlation was found between tongue surface temperature, perceived sweetness (*r* = 0.234, *p*-value = 0.002) and vanilla intensity (*r* = 0.226, p-value = 0.003). Perceived sweetness intensity was significantly higher for warm custard (tongue = 37.3 °C, sweetness = 20.5) than frozen custard (tongue = 27.1 °C, sweetness = 13.3). This suggests that temperature changes on the tongue during food consumption could significantly contribute to the perceived intensity of sweetness. The findings provide valuable insights to food industries interested in sugar reduction.

Video

Video to this article can be found online at https://doi.org/10.1016/j.sctalk.2025.100424.

**Figures and tables**

**Fig. 1:**
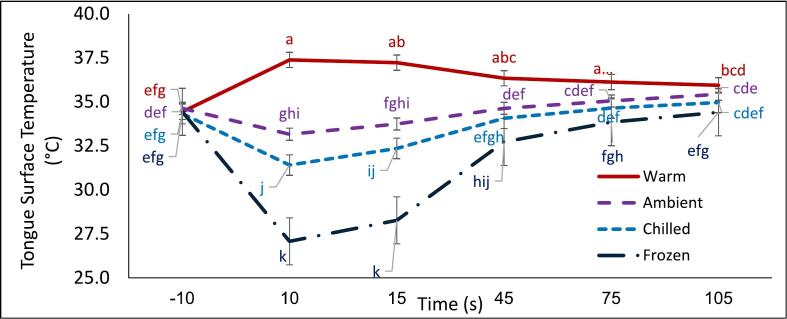
Interaction plot showing mean ± standard error for tongue surface temperature (°C) for custard served at different temperatures at various timepoints. (For interpretation of the references to color in this figure legend, the reader is referred to the web version of this article.) Serving temperatures include warm (red); ambient (purple); chilled (light blue); frozen (dark blue); Timepoints include -10s (baseline), 10s (sample in-mouth), 15 s (immediately post swallowing) and after swallowing timepoints 45 s, 75 s and 105 s. Different letters indicate significant differences at p < 0.05.

**Fig. 2:**
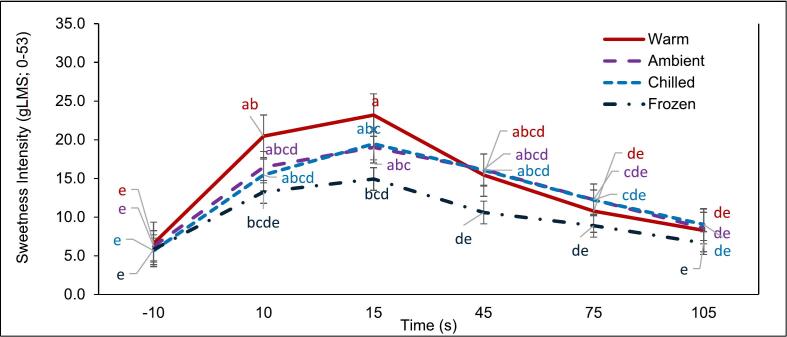
Interaction plot showing mean ± standard error for sweetness intensity ratings for custard served at different temperatures at various timepoints. (For interpretation of the references to color in this figure legend, the reader is referred to the web version of this article.) Serving temperatures include warm (red); ambient (purple); chilled (light blue); frozen (dark blue); Timepoints includes -10s (baseline), 10s (sample in-mouth), 15 s (immediately post swallowing) and after swallowing timepoints 45 s, 75 s and 105 s. Different letters indicate significant differences at p < 0.05.

**Fig. 3:**
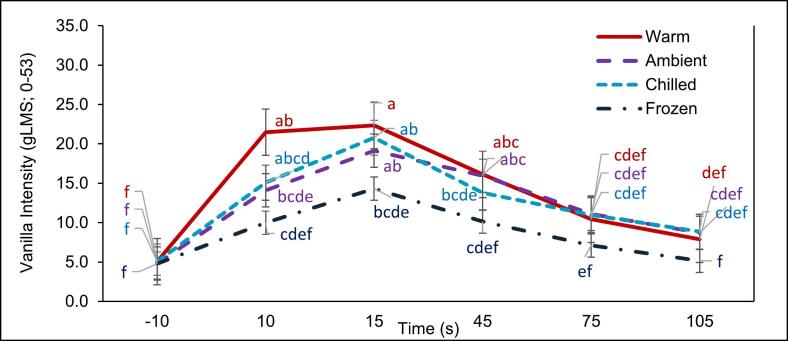
Interaction plot showing mean ± standard error for vanilla intensity ratings for custard served at different temperatures at various timepoints. Serving temperatures include warm (red); ambient (purple); chilled (light blue); frozen (dark blue); Timepoints includes -10s (baseline), 10s (sample in-mouth), 15 s (immediately post swallowing) and after swallowing timepoints 45 s, 75 s and 105 s. Different letters indicate significant differences at p < 0.05. (For interpretation of the references to colour in this figure legend, the reader is referred to the web version of this article.)

**Fig. 4:**
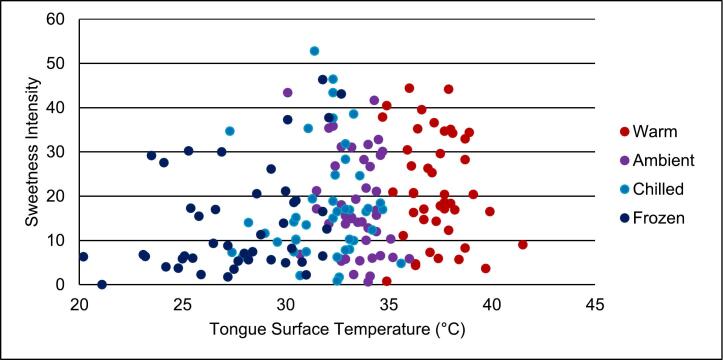
Correlation between tongue surface temperature and sweetness intensity at 10, 15 s. Serving temperatures include warm (red); ambient (purple); chilled (light blue); frozen (dark blue). Pearson correlation test found a weak positive correlation (r = 0.234; p-value = 0.002). (For interpretation of the references to colour in this figure legend, the reader is referred to the web version of this article.)

**Fig. 5:**
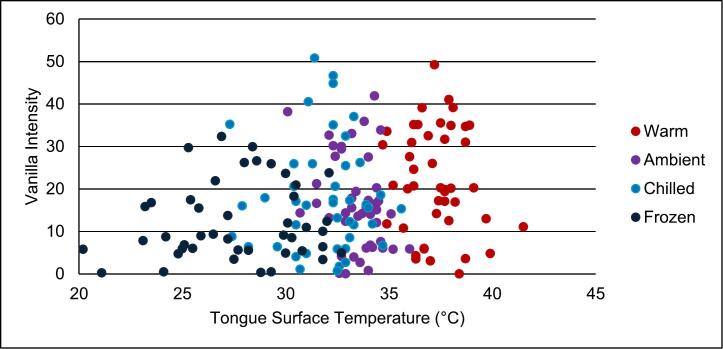
Correlation between tongue surface temperature and vanilla intensity at 10, 15 s. Serving temperatures include warm (red); ambient (purple); chilled (light blue); frozen (dark blue). Pearson correlation test found a weak positive correlation (r = 0.226; p-value = 0.003). (For interpretation of the references to colour in this figure legend, the reader is referred to the web version of this article.)

## CRediT authorship contribution statement

**Hannah McNeill:** Conceptualization, Formal analysis, Investigation, Methodology, Visualization, Writing – original draft. **Rebecca Ford:** Supervision, Writing – review & editing. **Ian Fisk:** Resources, Supervision. **Margaret Thibodeau:** Methodology, Resources, Supervision, Writing – review & editing. **Gloria Liu:** Resources, Supervision, Writing – review & editing. **Marion Doyennette:** Resources, Supervision, Writing – review & editing. **Qian Yang:** Conceptualization, Formal analysis, Funding acquisition, Methodology, Supervision, Visualization, Writing – original draft, Writing – review & editing.

## Data Availability

Data will be made available on request.
